# Effects of moderate-protein and high-protein diets, including mealworm meal or poultry by-product meal, on immunological variables in healthy adult dogs

**DOI:** 10.1186/s12917-025-04945-z

**Published:** 2025-08-05

**Authors:** Sophie-Kristin Heinze, Jürgen Zentek, Kathrin Büttner, Andreas Moritz, Nadine Paßlack

**Affiliations:** 1 Clinic for Small Animals, Internal Medicine, Faculty of Veterinary Medicine, Justus-Liebig-University Giessen, Giessen, Germany; 2 Chair for Animal Nutrition and Dietetics, Department of Veterinary Sciences, Faculty of Veterinary Medicine, Ludwig-Maximilians-Universität München, Munich, Germany; 3 Institute of Animal Nutrition, Department of Veterinary Medicine, Freie Universität Berlin, Berlin, Germany; 4 Unit for Biomathematics and Data Processing, Justus-Liebig-University Giessen, Giessen, Germany

**Keywords:** Canine, Insect, Diet, T helper cells, Cytotoxic T cells, Immunoglobulins, Blood count

## Abstract

**Background:**

Insect meals are increasingly being used as an ingredient in diets for dogs. However, little is known about their effects on the immune function of the animals. In the present investigation, mealworm meal was included in two complete diets with either a moderate or a high protein concentration (3.47% or 5.45% nitrogen in dry matter). Two diets with comparable protein levels (3.66% and 5.17% nitrogen in dry matter), but based on poultry by-product meal, served as control treatments. The diets were offered to 10 healthy adult beagle dogs, using a randomized crossover design. Each diet was fed for 4 weeks. Fasting blood samples were collected on day 24 of each feeding period for immune cell phenotyping, proliferation and phagocytosis assays, as well as for the measurement of plasma immunoglobulin (Ig) concentrations.

**Results:**

All dogs remained healthy throughout the study. The percentages of CD4^+^ cells in the blood of the dogs were lower, and the percentages of CD8^+^ cells were higher, when the diets with mealworm meal and the high-protein diets were fed. An interaction effect between the dietary protein source and protein level could be detected for the phagocytic activity of blood granulocytes as well as for the plasma concentrations of IgA and IgE.

**Conclusions:**

Both the protein source and protein level had an impact on the immune system of the dogs. The observed immunological changes were, however, not linked to any adverse food reactions, suggesting that the clinical relevance of these findings is likely small. Further studies should evaluate the immunological properties of dietary mealworm meal also in diseased animals, particularly in allergic dogs.

## Background

Insects are increasingly being used as a new or alternative protein source within the pet food market [1]. According to European Union legislation, eight farmed insect species are currently permitted as processed animal protein in animal feed for this purpose: Black Soldier Fly (*Hermetia illucens*), Common Housefly (*Musca domestica*), Yellow Mealworm (*Tenebrio molitor*), Lesser Mealworm (*Alphitobius diaperinus*), House cricket (*Acheta domesticus*), Banded cricket (*Gryllodes sigillatus*), Field Cricket (*Gryllus assimilis*), and Silkworm (*Bombyx mori*) [2–4].

One advantage of insect-based diets is the low environmental footprint associated with the farming of insects [5]. When compared to other livestock, insects are more efficient in feed utilization [5, 6], have lower greenhouse gas emissions [5] and require less water and space [5, 7]. Besides environmental reasons, insect-based feed can also be an interesting dietary option in veterinary medicine. Insects might represent a “novel” protein source for many dogs and cats [8], and, thus, could be used in mono-protein diets for food-responsive or allergic animals [8–10]. On the other hand, especially data from human medicine also demonstrated an allergenic potential of edible insects [11, 12]. This risk has also been addressed for companion animals [13, 14].

To date, only a few studies have evaluated the influence of diets with insect protein on the immune system of dogs. In the investigation of Kröger et al. [15], diets based on either *Hermetia illucens* larvae meal or lamb meal were fed to healthy adult beagle dogs. The authors found no differences in the complete and differential blood count, specific immune cell subpopulations in the blood (CD3^+^, CD4^+^, CD5^+^, CD8ß^+^, CD21^+^, MHC II^+^) or in the in vitro proliferative activity of blood leukocytes between the feeding groups. In line, Seo et al. [16] did not detect any changes in the serum levels of immunoglobulin (Ig)G, interleukin-10 or tumor necrosis factor-alpha, when senior dogs were fed either a control diet based on poultry meal and rice powder or a comparable diet but with 5% black soldier fly larvae. In addition, most values of the complete and differential blood count did not differ between the groups. The only exception was formed by the white blood cell counts, which were within the physiological reference range, but slightly higher in the group receiving the black soldier fly larvae [16]. The binding of storage mite-specific serum IgE to mealworm protein has been demonstrated by Western Blot analyses [13]. Based on the results, the investigators assumed a potential risk for cross-reactivity between mealworm and mite protein in allergic patients [13]. In this context, pan-allergens present in insects and arthropods (including mites), such as tropomyosin, have already been investigated in human medicine [17, 18] and described as a notable risk for allergic reactions in sensitized individuals [19, 20].

Only two studies are available on the effects of insect-based diets in dogs suffering from a food allergy so far. A commercial diet with mealworm meal was evaluated in atopic dogs with diagnosed adverse food reactions. While skin lesions were significantly improved by the diet (*P* < 0.05), pruritus was only reduced in 8 out of 15 dogs (*P* > 0.05), and the coat quality was unaffected in the majority of the animals (*P* < 0.05) [21]. A recent case report finally described the successful use of a diet with black soldier fly larvae meal in a dog with gastrointestinal symptoms of a food allergy [9].

Overall, it can be assumed that insect-based diets have an immunomodulatory potential in dogs. However, detailed mechanistic studies are limited so far. It was the aim of the present study to investigate the immunological effects of a diet containing mealworm meal at different inclusion levels in healthy adult dogs. Control diets based on poultry by-product meal served as a reference.

## Methods

The study was approved by the relevant authority in Giessen, Germany (Regierungspräsidium Gießen; approval number G 33/2022).

### Study design

Ten healthy research dogs (six male neutered and four female neutered beagles, born in the European Union at a registered breeder, aged 33 ± 11 months at the beginning of the trial, initial body weight 9.37 ± 1.41 kg) were selected based on their availability for the present study. The animals were housed in the experimental facilities of the Clinic for Small Animals of the Justus-Liebig-University Giessen. They were kept indoors as pairs and had daily access to an outdoor area as well as daily walks.

Four experimental diets were prepared for the investigation, differing in the protein source and level. Two diets had a moderate protein concentration (3.66 and 3.47% nitrogen (N) in dry matter (DM)), and the other two diets had a high protein concentration (5.17 and 5.45% N in DM). At each protein level, one food was based on poultry by-product meal, and the other one on mealworm meal. A complete replacement of poultry by-product meal by mealworm meal was considered instead of a partial replacement, as this approach allowed for a more focused evaluation of the respective meal, particularly by avoiding potential interfering effects between these protein sources if included in the same diet.

The composition and analyzed nutrient concentrations of the diets are presented in Tables 1, 2 and 3. For the conversion of the analyzed N concentration to crude protein, it had been taken into account that the protein sources of the diets differed. A conversion factor of 6.25 is generally used; however, it is assumed that this factor overestimates the protein content of insects due to nonprotein N derived from chitin of their exoskeleton [22–24]. Thus, different conversion factors are recommended for specific insect species [22–24]. In the present study, the diets with mealworm meal included further ingredients that also contributed to the total N concentration, such as rice flour and blood meal. As a consequence, different N-to-protein conversion factors were applied for the same diet, considering (a) the dietary composition, (b) the N concentrations of the ingredients, and (c) the relative contribution of the ingredients to the total N concentration of the diet.

**Table 1:** Composition (%) of the experimental diets

	Moderate-protein diets		High-protein diets	
	PBM	MWM	PBM	MWM
Rice flour	64.7	65.0	52.4	53.1
Poultry by-product meal	17.4	-	37.4	-
Mealworm meal	-	15.1	-	32.4
Rapeseed oil	4.50	5.36	2.00	3.83
Cellulose	5.44	3.01	5.35	0.21
Cod liver oil	0.60	0.57	0.63	0.58
Titanium dioxide	0.24	0.23	0.26	0.24
Calcium carbonate	0.98	2.03	-	2.23
Monocalcium phosphate	1.36	2.24	-	1.81
Blood meal	2.17	3.95	-	3.72
Minerals and vitamins	2.61	2.51	1.96	1.88

PBM: Poultry by-product meal; MWM: mealworm meal

**Table 2:** Analyzed dry matter, crude nutrient, and mineral concentrations of the experimental diets

	Moderate-protein diets		High-protein diets	
	PBM	MWM	PBM	MWM
Dry matter (g/100 g)	92.8	93.6	94.3	93.9
***In g/100 g dry matter***				
Nitrogen	3.66	3.47	5.17	5.45
Crude protein^1^	22.9	20.5	32.3	31.4
Crude fat	8.30	7.67	8.40	8.33
Crude fiber	3.21	3.65	3.59	3.73
Crude ash	6.27	5.74	5.58	5.52
Calcium	1.30	1.19	1.21	1.15
Phosphorus	0.79	0.74	0.78	0.69
Sodium	0.20	0.17	0.17	0.18
Potassium	0.37	0.36	0.28	0.41
Magnesium	0.17	0.16	0.16	0.18
***In mg/100 g dry matter***				
Copper	2.65	2.12	1.04	2.06
Zinc	17.3	15.5	15.9	15.6
Iron	25.9	29.0	16.2	30.1
Manganese	4.84	5.41	3.59	4.01

^1^ Calculated (see Material and Methods)

PBM: Poultry by-product meal; MWM: mealworm meal

**Table 3:** Analyzed amino acid concentrations of the experimental diets

	Moderate-protein diets		High-protein diets	
	PBM	MWM	PBM	MWM
**In g/100 g dry matter**				
Taurine	0.04	0.16	0.08	0.14
Aspartic acid	0.80	0.90	1.10	1.27
Threonine	0.77	0.71	1.15	1.16
Serine	1.27	0.97	1.88	1.53
Glutamic acid	2.80	2.50	4.00	3.76
Glycine	1.82	1.08	2.68	1.75
Alanine	1.45	1.51	1.98	2.42
Valine	1.45	1.55	1.94	2.40
Isoleucine	0.92	0.86	1.45	1.56
Leucine	2.06	2.14	2.63	3.31
Tyrosine	0.75	1.04	0.90	1.96
Phenylalanine	1.05	0.96	1.37	1.39
Histidine	0.49	0.74	0.49	1.11
Lysine	1.08	1.22	1.45	1.96
Arginine	1.51	1.24	2.22	2.14
Proline	1.50	1.17	2.29	2.01
Methionine	0.36	0.29	0.46	0.44
Cystine	0.43	0.24	0.63	0.35
Hydroxyproline	0.00	0.00	0.00	0.00

PBM: Poultry by-product meal; MWM: mealworm meal

Before the study, the dogs were never fed a diet containing mealworm meal, but commercial diets containing poultry protein. Therefore, the diets with mealworm meal included a “new” protein, and the control diets based on poultry by-product meal included a “known” protein for the immune system of the animals.

The diets were offered in a randomized crossover trial, where the dogs in one kennel received the same diet. Deionized water was available *ad libitum* throughout the study. The diets were produced as a homogeneous meal, but mixed with a defined amount of deionized water before feeding. The individual amounts of food were calculated to maintain the body weight of the dogs [25]. The animals were fed twice a day. Remainders were weighed back to calculate the daily food intake. The body weight of the dogs was recorded weekly.

Each diet was fed for four weeks. Fasting blood samples were taken on day 24 by puncture of the *Vena cephalica antebrachii* or the *Vena saphena lateralis*.

### Feed analysis

The dry matter, crude nutrient, and mineral concentrations of the experimental diets were determined according to the official methods for feed analysis [26]. For the crude fat measurements, the method was modified as described before [27]. The amino acid concentrations in the diets were analyzed using the Biochrom 20 Plus (Amersham Pharmacia Biotech, Piscataway, USA). More details are provided elsewhere [28].

### Phenotyping of peripheral blood mononuclear cells

For the phenotyping, blood collected in lithium heparin tubes was used (approximately 4.9 mL per dog). In a first step, the blood was diluted 1:2 with phosphate-buffered saline without calcium, magnesium, and phenol red (PBS, Capricorn Scientific GmbH, Ebsdorfergrund, Germany) in a new 15 mL tube (Greiner Bio-One GmbH, Frickenhausen, Germany). The mixture was carefully pipetted onto 3 mL Biocoll^®^ separation solution (density 1.077 g/mL; Capricorn Scientific GmbH, Ebsdorfergrund, Germany). The tubes were then centrifuged at 800 x *g* and room temperature for 20 min (Heraeus Megafuge 16R, Thermo-Fisher Scientific, Karlsruhe, Germany). Afterwards, the supernatant was carefully removed to allow for a collection of the so-called “interphase” (phase between the supernatant and Biocoll^®^, containing the peripheral blood mononuclear cells). The interphase was pipetted into a new 15 mL tube with 10 mL ice-cold PBS. After centrifugation at 400 x *g* and 4 °C for 10 min, the supernatant was removed, and the pellet was resuspended in 10 mL ice-cold PBS. The tubes were centrifuged at 300 x *g* and 4 °C for 10 min. The supernatant was removed, and the pellet was resuspended in 1 mL of cell culture medium. The cell culture medium was composed of RPMI 1640 Medium with L-Glutamine (Capricorn Scientific GmbH, Ebsdorfergrund, Germany), 10% Fetal Bovine Serum (FBS, Capricorn Scientific GmbH, Ebsdorfergrund, Germany) and 1% Penicillin-Streptomycin (Sigma-Aldrich Chemie GmbH, Taufkirchen, Germany).

In the next step, 10 µL of the cell suspension were mixed with 90 µL of trypan blue (Sigma-Aldrich Chemie GmbH, Taufkirchen, Germany) to allow for a cell counting in a Neubauer counting chamber, using a microscope with a 10x objective (either Motic Europe S.L.U., Barcelona, Spain or Leitz Photographica Auction, Vienna, Austria). The number of vital cells in the four main squares was counted, averaged, and multiplied by the dilution factor of trypan blue (10). Finally, this number had to be multiplied by 10^4^ to receive the number of cells/mL, as the volume of the Neubauer counting chamber was 0.1 µL (10^4^ × 0.1 µL = 1 mL).

Next, the cell suspension was adjusted to a cell density of 1 × 10^7^/mL, using the cell culture medium described above. Of this cell suspension, 100 µL each (corresponding to 1 × 10^6^ cells) was pipetted into 5 tubes (VWR^®^, Test Tubes, 5 mL, polystyrene, VWR International GmbH, Darmstadt) per dog. The remaining 500 µL of the cell suspension were used for the proliferation assay (see 2.4.). All tubes were kept on ice.

Fifty µL of an antibody (anti Dog CD3:FITC/CD4:RPE/CD8:Alexa Fluor^®^ 647 (Bio-Rad Laboratories GmbH, Feldkirchen, Germany), Mouse anti Canine CD21:RPE (Bio-Rad Laboratories GmbH, Feldkirchen, Germany), Mouse anti Human CD14:FITC (Bio-Rad Laboratories GmbH, Feldkirchen, Germany), MHC Class II rat monoclonal antibody, FITC (OriGene Technologies, Inc., Rockville, MD, USA)) were pipetted into the tubes with the cell suspension, resulting in 4 different treatments per dog. While the MHC II antibody was used undiluted, the other antibodies were diluted 1:10 with fluorescence-activated cell sorting (FACS) buffer. This buffer consisted of PBS and 0.5% bovine serum albumin (BSA, Carl Roth GmbH + Co. KG, Karlsruhe, Germany) or 2.5% FBS. The fifth tube with cell suspension was used as a negative control without an antibody to determine the autofluorescence of the cells. Instead of an antibody, 50 µL of PBS was pipetted into this tube.

The prepared tubes were vortexed, placed on ice, and incubated for 25 min. Afterwards, 2 mL FACS buffer was pipetted into the tubes, which were then centrifuged at 389 x *g* and 4 °C for 5 min. The supernatant was discarded, and the cell pellet resuspended with 300 µL FACS buffer. The samples were assigned to flow cytometric measurements, using the Accuri™ C6 Plus System (BD Bioscience, Franklin Lakes, New Jersey, USA).

### Mitogen-stimulated proliferative activity of blood lymphocytes

For the mitogen-induced proliferation assay, the remaining cell suspension (see 2.3.) was adjusted to a cell density of 4 × 10^6^/mL, using the described cell culture medium.

In a first step, 100 µL of pre-diluted mitogens (pokeweed mitogen (PWM), concanavalin A (ConA), phytohemagglutinin, M form (PHA-M), all Sigma-Aldrich Chemie GmbH, Taufkirchen, Germany) were added into the wells of a cell culture microplate (96 wells, u-bottom, sterile, CELLSTAR^®^ TC, Greiner Bio-One GmbH, Frickenhausen, Germany). Afterwards, 100 µL of the cell suspension (corresponding to 4 × 10^5^ cells) was added into the wells. As a negative control, 100 µL of cell culture medium was mixed with 100 µL of cell suspension. The final concentration of the mitogens in the wells was 2.5 µg PWM/mL, 5.0 µg ConA/mL and 10 µg PHA-M/mL. The cell culture plate was incubated in a CO_2_-incubator (37 °C, 5% CO_2_, 92.7% humidity; Memmert GmbH + Co. KG, Schwabach, Germany) for 48 h.

Next, 60 µM 5-Bromo-2′-deoxyuridine (BrdU; Sigma-Aldrich Chemie GmbH, Taufkirchen, Germany) was pipetted into each well, followed by an incubation of the cell culture plate in the CO_2_-incubator for 24 h. After the incubation, the content of the wells was transferred into 5 mL tubes (VWR International GmbH, Darmstadt). Wells with the same condition (negative control, PWM, ConA, PHA-M) were pooled for each animal, resulting in 4 tubes per dog. The tubes were centrifuged at 423 x *g* and 4 °C for 5 min. The supernatant was removed, and 2 mL of FACS buffer was added to the tubes afterwards. The tubes were again centrifuged at 423 x *g* and 4 °C for 5 min. After discarding the supernatant, the cell pellet was resuspended in 1 mL FACS buffer. Then, 250 µL of “Inside Fix” (Cell Signaling Buffer A, Miltenyi Biotec B.V. & CO. KG, Bergisch Gladbach, Germany) was pipetted into the tubes. The tubes were vortexed and incubated at room temperature for 15 min. Subsequently, the tubes were centrifuged at 368 x *g* and 4 °C for 5 min. The supernatant was removed, and 1 mL of ice-cold methanol was carefully pipetted into the tubes. The tubes were vortexed for 2 s and incubated on ice for 30 min. Then, 2.5 mL of FACS buffer was added, the tubes were centrifuged at 368 x *g* and 4 °C for 5 min, and the supernatant was discarded. Two washing steps were followed, where 3.5 mL FACS buffer was pipetted into the tubes, which were then centrifuged at 368 x *g* and 4 °C for 5 min, and the supernatant was removed. Afterwards, 250 µL deoxyribonuclease I from bovine pancreas (DNAse I; Sigma-Aldrich Chemie GmbH, Taufkirchen, Germany) was added to the tubes. The DNAse I was pre-diluted 1:10 with DNAse buffer (New England BioLabs GmbH, Frankfurt, Germany). After an incubation at 37 °C for 30 min in a water bath, 2 mL FACS buffer was pipetted into the tubes, followed by another centrifugation at 368 x *g* and 4 °C for 5 min. The supernatant was removed, and the pellet was resuspended with 10 µL FITC Mouse Anti-BrdU (BD Pharmingen™ FITC Mouse Anti-BrdU Set, BD Biosciences, Franklin Lakes, New Jersey, USA). This was followed by an incubation on ice for 30 min and protected from light. Subsequently, 2 mL FACS buffer was added, and the tubes were centrifuged at 368 x *g* and 4 °C for 5 min. The supernatant was discarded, and the cell pellet resuspended in 300 µL FACS buffer, followed by the flow cytometric measurements (Accuri™ C6 Plus System, BD Bioscience, Franklin Lakes, New Jersey, USA).

Finally, the proliferation index (%) was calculated as follows: proliferative activity of mitogen-stimulated lymphocytes (% gated) divided by the proliferative activity of unstimulated lymphocytes (% gated).

### Phagocytosis assay

A commercial test kit (IngoFlowEx Kit, EXBIO Praha, a.s., Vestec, Czech Republic) was used for the measurement of the phagocytic activity of blood monocytes and granulocytes. For each dog, 3 tubes (VWR^®^, Test Tubes, 5 mL, polystyrene, VWR International GmbH, Darmstadt) with 100 µL lithium-heparin blood were prepared. The tubes were incubated on ice for 10 min. Then, 20 µL of a FITC-labeled *E. coli* solution was pipetted into the tubes. Two tubes per dog were incubated in a water bath at 37 °C for 10 min, while the third tube remained on ice (“ice control”). This was followed by another incubation of all tubes on ice for 5 min. Subsequently, 100 µL “Quenching Solution” was added to the tubes, which were then vortexed and placed on ice again. After the addition of 3 mL “Wash Buffer”, the tubes were centrifuged at 270 x *g* and 4 °C for 5 min. The supernatant was discarded, followed by 2 more washing steps with 3 mL “Wash Buffer”, centrifugation at 270 x *g* and 4 °C for 5 min, and removal of the supernatant. Then, 2 mL “Lysing Solution” was added, and the tubes were incubated at room temperature and under light protection for 20 min. After centrifugation at 270 x *g* and 4 °C for 5 min, the supernatant was discarded. Two washing steps were followed with 3 mL “Wash Buffer”, centrifugation at 270 x *g* and 4 °C for 5 min, and removal of the supernatant. Then, 300 µL “DNA Staining Solution” was pipetted into the tubes. After an incubation on ice for 10 min, the samples were measured using flow cytometry (Accuri™ C6 Plus System, BD Bioscience, Franklin Lakes, New Jersey, USA).

### Immunoglobulin concentrations in the plasma

The concentrations of IgG, IgA, IgM, and IgE in the plasma of the dogs were measured with commercial test kits (Dog IgG ELISA Kit # E-40G, Dog IgA ELISA Kit # E-40 A, Dog IgM ELISA Kit # E-40 M and Dog IgE ELISA Kit # E-40E, all ICL, Inc., Portland, OR, USA), following the instructions of the manufacturer.

### Blood count and plasma urea and creatinine concentrations

Whole EDTA blood of the dogs was examined for a complete and differential blood count, using the ADVIA 2020i (Siemens Healthineers AG) of the central laboratory of the Clinic for Small Animals, internal medicine, of the Justus-Liebig-University Giessen. The urea and creatinine concentrations in lithium heparin plasma of the animals were analyzed in the same laboratory with the ABX Pentra 400 (Horiba Europe GmbH).

### Statistical data analysis

**Fig. 1:**
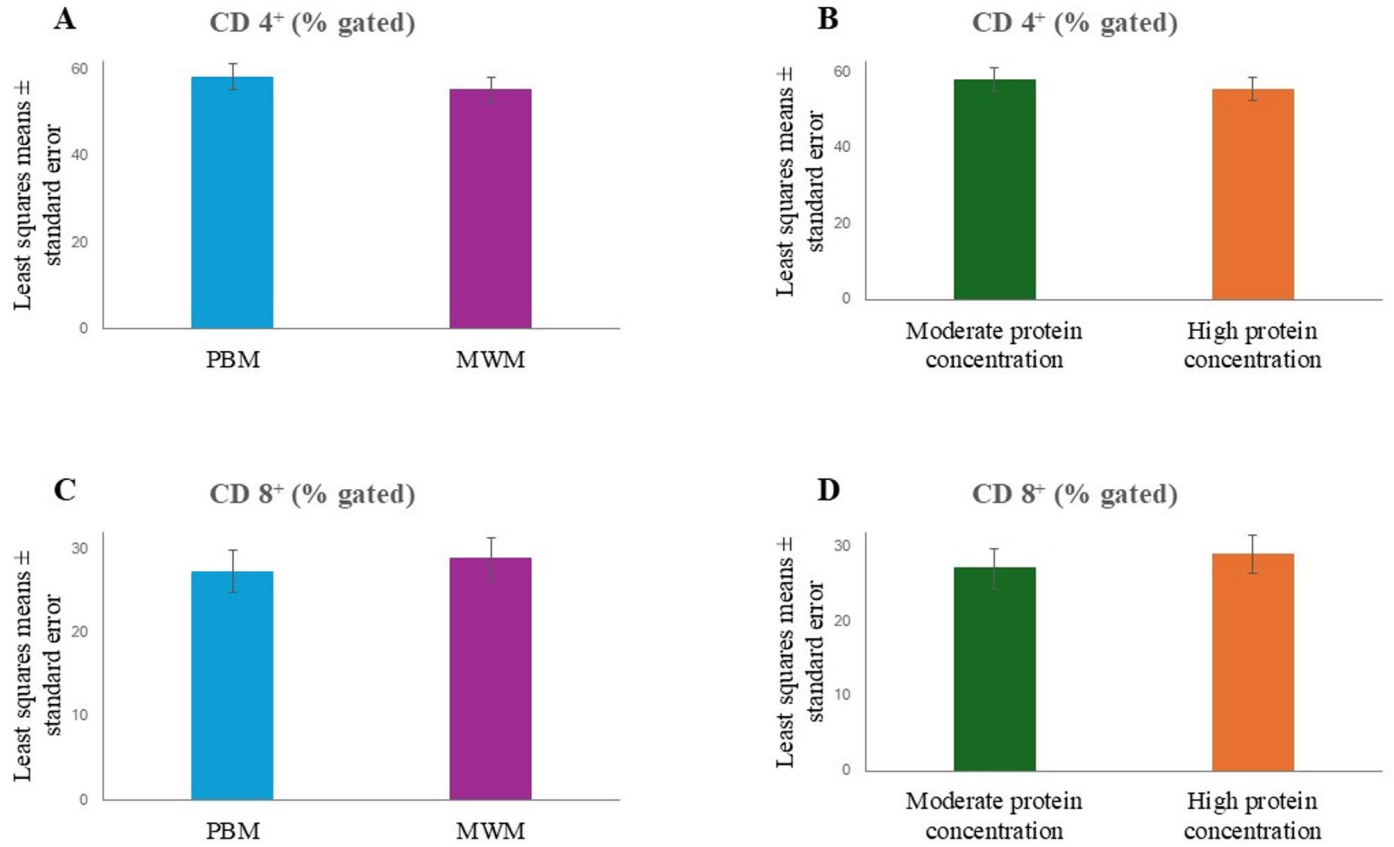
**A**: Percentage of CD4^+^ cells in the blood of dogs fed diets with poultry by-product meal (PBM) or mealworm meal (MWM). **B**: Percentage of CD4^+^ cells in the blood of dogs fed moderate-protein or high-protein diets. **C**: Percentage of CD8^+^ cells in the blood of dogs fed diets with PBM or MWM. **D**: Percentage of CD8^+^ cells in the blood of dogs fed moderate-protein or high-protein diets. Data were analyzed together for the 2 groups receiving PBM and MWM (**A**, **C**) and for the 2 groups receiving the moderate-protein and high-protein diets (**B**, **D**)

The data were analyzed with SAS 9.4 (SAS^®^ Institute Inc., 2013. Base SAS^®^ 9.4 Procedures Guide: Statistical Procedures, 2nd edition ed. Statistical Analysis System Institute Inc., Cary, NC, USA), and Fig. 1 was created with Microsoft Excel (Microsoft Corporation). A repeated measures ANOVA was used to assess the fixed effects of the dietary protein source (poultry by-product meal, mealworm meal), protein level (moderate, high), and their interaction. In the case of a significant main effect, Bonferroni-adjusted pairwise comparisons were carried out. A *P* value < 0.05 was considered to be statistically significant. All data in the tables are presented as least squares means ± standard error.

## Results

### Feed intake, body weight, and animal health

The moderate-protein diet based on poultry by-product meal was refused by four dogs. These animals had to be excluded from the ongoing feeding period and were not considered for the respective data set. The other experimental diets were well accepted and tolerated by all dogs. The feed intake (g DM/day) was slightly lower when the diets with poultry by-product meal were fed (*P* < 0.05; Table 4). When the DM intake was calculated per kg body weight of the dogs, an effect of the dietary protein source was not detectable (*P* > 0.05); however, during the sampling periods, the feed intake (g DM/kg body weight/day) was slightly lower when the high-protein diets were offered (*P* = 0.021). The body weight of the dogs was higher when the diets with mealworm meal and the diets with a higher protein level were fed (*P* < 0.05).

**Table 4:** Feed intake and body weight (BW) of dogs fed moderate-protein and high-protein diets with either poultry by-product meal (PBM) or mealworm meal (MWM). Least squares means ± standard error

	Moderate-protein diets						High-protein diets						*P* value		
	PBM (*n* = 6)			MWM (*n* = 10)			PBM (*n* = 10)			MWM (*n* = 10)			Protein source	Protein level	Protein source x protein level
**Per feeding period**															
Feed intake (g DM/day)	183	±	7.87	198	±	7.43	187	±	7.43	195	±	7.43	0.008	0.685	0.482
Feed intake (g DM/kg BW/day)	20.6	±	0.64	21.1	±	0.54	19.8	±	0.54	20.4	±	0.54	0.156	0.162	0.885
BW (kg)	8.89	±	0.46	9.50	±	0.45	9.53	±	0.45	9.62	±	0.45	0.035	0.020	0.264
***Per sample collection period (=last week of a feeding period)***															
Feed intake (g DM/day)	184	±	8.71	198	±	7.97	186	±	7.97	194	±	7.97	0.047	0.565	0.618
Feed intake (g DM/kg BW/day)	21.6	±	0.72	20.9	±	0.61	19.7	±	0.61	19.8	±	0.61	0.780	0.021	0.579
BW (kg)	8.56	±	0.46^a^	9.57	±	0.45^b^	9.55	±	0.45^b^	9.80	±	0.45^b^	< 0.001	< 0.001	0.035

DM: dry matter

Different superscript letters in the same row indicate a significant group difference (*P* < 0.05)

The dogs remained healthy throughout the study, as also emphasized by the complete and differential blood count and clinical chemistry values, which were within the physiological reference range for dogs (except for minimally lowered blood leukocytes in the group receiving the mealworm meal containing diet with a moderate protein level; Table 5). However, some group differences in those blood parameters were observed. Dogs fed the diets with mealworm meal showed lower numbers of thrombocytes (*P* = 0.008), leukocytes (*P* = 0.002), neutrophilic granulocytes (*P* = 0.005), and lymphocytes (*P* = 0.005), but higher numbers of eosinophilic granulocytes (*P* = 0.005) than the control group. Moreover, the number of erythrocytes (*P* = 0.037) in the blood of the animals was lower, and the plasma concentrations of urea (*P* = 0.001) were higher, when the dogs received the high-protein diets.

**Table 5:** Complete and differential blood count as well as plasma urea and creatinine concentrations of dogs fed moderate-protein and high-protein diets with either poultry by-product meal (PBM) or mealworm meal (MWM). Least squares means ± standard error

	Moderate-protein diets						High-protein diets						*P* value		
	PBM (*n* = 6)			MWM (*n* = 10)			PBM (*n* = 10)			MWM (*n* = 10)			Protein source	Protein level	Protein source x protein level
Hemoglobin (mmol/L)	10.6	±	0.67	9.67	±	0.53	10.3	±	0.53	10.4	±	0.53	0.454	0.692	0.337
Hematocrit (L/L)	0.51	±	0.01	0.49	±	0.01	0.49	±	0.01	0.49	±	0.01	0.253	0.493	0.438
Thrombocytes (10^9^/L)	286	±	19.8	224	±	17.2	248	±	17.2	231	±	17.2	0.008	0.238	0.107
Reticulocytes (10^9^/L)	44.5	±	6.31	46.4	±	5.26	34.1	±	5.26	39.7	±	5.26	0.387	0.073	0.678
Erythrocytes (10^12^/L)	7.21	±	0.14	7.01	±	0.13	6.92	±	0.13	6.96	±	0.13	0.299	0.037	0.142
Leukocytes (10^9^/L)	6.75	±	0.33	5.45	±	0.28	5.87	±	0.28	5.49	±	0.28	0.002	0.064	0.070
Neutrophilic granulocytes (10^9^/L)	4.21	±	0.29	3.20	±	0.25	3.52	±	0.25	3.29	±	0.25	0.005	0.106	0.072
Basophilic granulocytes (10^9^/L)	0.05	±	0.01	0.03	±	0.01	0.04	±	0.01	0.04	±	0.01	0.156	0.696	0.339
Eosinophilic granulocytes (10^9^/L)	0.16	±	0.04	0.28	±	0.03	0.25	±	0.03	0.28	±	0.03	0.005	0.063	0.098
Lymphocytes (10^9^/L)	2.05	±	0.11	1.70	±	0.09	1.80	±	0.09	1.65	±	0.09	0.005	0.058	0.203
Monocytes (10^9^/L)	0.25	±	0.03	0.21	±	0.03	0.24	±	0.03	0.22	±	0.03	0.061	0.893	0.743
Urea (mmol/L)	4.35	±	0.65^a^	5.35	±	0.52^ab^	6.68	±	0.52^ab^	7.76	±	0.52^b^	0.064	0.001	0.935
Creatinine (µmol/L)	74.9	±	4.16	69.7	±	3.28	71.2	±	3.28	64.4	±	3.28	0.094	0.195	0.801

Reference values: hemoglobin (mmol/L): 8.06–12.21; hematocrit (L/L): 0.39–0.56; thrombocytes (10^9^/L): 150–500; reticulocytes (10^9^/L): 10–110; erythrocytes (10^12^/L): 5.64–8.30; leukocytes (10^9^/L): 5.48–13.74; neutrophilic granulocytes (10^9^/L): 2.78–8.73; basophilic granulocytes (10^9^/L): 0-0.11; eosinophilic granulocytes (10^9^/L): 0.00-1.47; lymphocytes (10^9^/L): 0.72–4.71; monocytes (10^9^/L): 0.06–0.83; urea (mmol/L): 3.3–9.82; creatinine (µmol/L): 53.00-122.00

Different superscript letters in the same row indicate a significant group difference (*P* < 0.05)

### Phenotyping of peripheral blood mononuclear cells

The phenotyping of peripheral blood mononuclear cells revealed lower percentages of CD4^+^ cells (T helper cells) in dogs receiving the diets with mealworm meal (*P* for the protein source = 0.007) as well as the high-protein diets (*P* for protein level = 0.019) (Table 6; Fig. 1). In contrast, the percentages of CD8^+^ cells (cytotoxic T cells) were higher when the diets with mealworm meal and the high-protein diets were fed (*P* = 0.044 and *P* = 0.018 for the protein source and protein level, respectively).

**Table 6:** Phenotyping of peripheral blood mononuclear cells of dogs fed moderate-protein and high-protein diets with either poultry by-product meal (PBM) or mealworm meal (MWM). Least squares means ± standard error

	Moderate-protein diets						High-protein diets						*P* value		
% gated	PBM (*n* = 6)			MWM (*n* = 10)			PBM (*n* = 10)			MWM (*n* = 10)			Protein source	Protein level	Protein source x protein level
CD4^+^	55.6	±	3.26	56.1	±	3.17	56.6	±	3.17	54.4	±	3.17	0.007	0.019	0.353
CD8^+^	29.4	±	2.65	28.3	±	2.60	28.6	±	2.60	29.3	±	2.60	0.044	0.018	0.239
CD4^+^CD8^+^	2.07	±	0.54	1.36	±	0.52	1.59	±	0.52	1.59	±	0.52	0.324	0.776	0.345
CD21^+^	11.9	±	1.46	12.5	±	1.20	12.3	±	1.20	11.3	±	1.20	0.882	0.725	0.405
CD14^+^	37.9	±	6.03	22.7	±	4.69	27.6	±	4.69	19.5	±	4.69	0.052	0.227	0.517
MHC II^+^	85.9	±	4.79	84.7	±	4.08	83.3	±	4.08	80.9	±	4.08	0.507	0.282	0.912

CD4^+^ and CD4^+^CD8^+^: T-helper cells; CD8^+^: cytotoxic T cells; CD21^+^: B cells; CD14^+^: myeloid cells; MHC II^+^: antigen-presenting cells

### Proliferation and phagocytosis assays

The mitogen-induced proliferation of lymphocytes isolated from the blood of the dogs was not affected by the dietary treatments (Table 7).

**Table 7:** Proliferation index (%) of blood leukocytes of dogs fed moderate-protein and high-protein diets with either poultry by-product meal (PBM) or mealworm meal (MWM). Least squares means ± standard error

	Moderate-protein diets						High-protein diets						*P* value		
Stimulating mitogens	PBM (*n* = 6)			MWM (*n* = 10)			PBM (*n* = 10)			MWM (*n* = 10)			Protein source	Protein level	Protein source x protein level
PWM	2.90	±	0.52	2.99	±	0.40	3.98	±	0.40	3.36	±	0.40	0.547	0.142	0.474
ConA	6.35	±	1.34	6.05	±	1.04	8.98	±	1.04	6.84	±	1.04	0.306	0.161	0.446
PHA-M	4.68	±	1.07	4.04	±	0.83	5.86	±	0.83	4.63	±	0.83	0.323	0.352	0.754

PWM: pokeweed mitogen, ConA: concanavalin A, PHA-M: phytohemagglutinin, M form

In addition, the in vitro phagocytosis of blood monocytes was not different among the groups (Table 8). For the phagocytic activity of blood granulocytes, an interaction effect between the dietary protein source and protein level could be detected (*P* = 0.040). The pairwise comparisons, however, could not further clarify the treatment effect (*P* > 0.05).

**Table 8:** Phagocytic activity of blood monocytes and granulocytes of dogs fed moderate-protein and high-protein diets with either poultry by-product meal (PBM) or mealworm meal (MWM). Least squares means ± standard error

	Moderate-protein diets						High-protein diets						*P* value		
% gated	PBM (*n* = 6)			MWM (*n* = 10)			PBM (*n* = 10)			MWM (*n* = 10)			Protein source	Protein level	Protein source x protein level
Monocytes	63.5	±	4.67	55.6	±	3.67	57.2	±	3.67	58.5	±	3.67	0.363	0.613	0.244
Granulocytes	77.3	±	3.73	64.9	±	3.13	65.8	±	3.13	70.7	±	3.13	0.187	0.268	0.040

### Plasma immunoglobulin concentrations

The Ig concentrations in the plasma of the dogs were largely unaffected by the different dietary treatments (Table 9). An interaction effect between the dietary protein source and protein level could be detected for the IgA concentrations (*P* = 0.043) and IgE concentrations (*P* = 0.044). However, when pairwise comparisons among the groups were carried out, no significant differences were observed (*P* > 0.05).

**Table 9:** Immunoglobulin (Ig) concentrations in the plasma of dogs fed moderate-protein and high-protein diets with either poultry by-product meal (PBM) or mealworm meal (MWM). Least squares means ± standard error

	Moderate-protein diets						High-protein diets						*P* value		
	PBM (*n* = 6)			MWM (*n* = 10)			PBM (*n* = 10)			MWM (*n* = 10)			Protein source	Protein level	Protein source x protein level
IgG (mg/mL)	142	±	19.5	154	±	15.1	128	±	15.1	146	±	15.1	0.379	0.536	0.867
IgA (mg/mL)	6.27	±	0.95	5.23	±	0.75	4.72	±	0.75	7.43	±	0.75	0.307	0.721	0.043
IgM(mg/mL)	5.53	±	0.67	4.51	±	0.52	4.18	±	0.52	3.83	±	0.52	0.294	0.125	0.636
IgE (µg/mL)	6.53	±	2.05	5.67	±	2.02	5.56	±	2.02	7.36	±	2.02	0.166	0.242	0.044

## Discussion

Different insect species can serve as an ingredient for pet food. Currently, the most frequently processed insects are larvae of the Black Soldier Fly (*Hermetia illucens*), Yellow Mealworms (*Tenebrio molitor*), and House crickets (*Acheta domesticus*) [29]. Published studies on the evaluation of insect-based diets in dogs particularly focused on the use of *Hermetia illucens* larvae meal and its effects on the feed acceptance, feed tolerance, apparent nutrient digestibility and the gut microbiome [15, 30–35]. In the study of Kröger et al. [15], immune variables were also measured.

Although pet food that includes mealworm meal is commercially available, study results on its metabolic and immunological effects remain scarce. Zhang et al. [36] evaluated the apparent nutrient digestibility of diets with different protein sources in dogs. They found a lower apparent crude protein and crude fat digestibility when mealworm meal was included in the diet instead of some other meat meals [36]. Atopic dogs with adverse food reactions fed a diet based on mealworm meal showed an improvement of certain dermatological parameters, while others were unaffected. The diet was largely well tolerated by the dogs; however, one dog developed acute gastrointestinal symptoms and had to be removed from the trial [21].

In the present investigation, the immunomodulatory effects of diets with two different inclusion levels of mealworm meal were evaluated in healthy dogs. As control treatments, diets with comparable nitrogen levels but based on poultry by-product meal were used. The results demonstrated that the diets with mealworm meal partly influenced the cellular and humoral immune responses of the animals.

This was reflected by lower numbers of blood leukocytes, neutrophilic granulocytes, and lymphocytes in the dogs receiving the diets with mealworm meal. For data interpretation, however, it should be considered that all values remained within the physiological reference range. Moreover, the percentage of T helper cells (CD4^+^) in the blood of the dogs was lower, and the percentage of cytotoxic T cells (CD8^+^) was higher when the diets with mealworm meal were fed. In general, both T helper cells and cytotoxic T cells are important effector cells of the adaptive immune system [37]. While CD4^+^ cells recognize antigens presented by MHC II molecules, CD8^+^ cells are MHC-class I-restricted [38]. CD4^+^ cells can differentiate into a variety of subpopulations, which exert distinct functionalities [37, 39].

Several studies have demonstrated effects on the distribution of CD4^+^ and CD8^+^ cells in the blood of dogs. Besides an impact of dietary factors, such as yeast cell wall components [40, 41], beta-carotene [42], lutein [43], or fatty acids and vitamin E [44], especially dogs with atopic dermatitis are the focus of ongoing research. Canine atopic dermatitis is a hereditary, multifactorial disease with an underlying immune response to a variety of potential allergens [45]. In this context, changes in the percentages of CD4^+^ and CD8^+^ cells in the blood of atopic dogs have been reported. While some authors observed an increase in CD4^+^ cells and a decrease in CD8^+^ cells [46], others found an increase in CD8^+^ cells, while the percentage of CD4^+^ cells was unaffected [47–49]. Moreover, a few studies did not reveal a difference in the CD4^+^/CD8^+^ ratio in the blood of healthy control and atopic dogs [50, 51]. Nevertheless, when summarizing the available data [46–49], it can be concluded that immune variables of atopic dogs were often altered compared to healthy individuals. Concerning the present results, one might therefore speculate if the observed differences in the percentages of T helper cells and cytotoxic T cells in the blood of the dogs could represent a kind of allergic response to the dietary treatment. The observed higher numbers of eosinophilic granulocytes measured in the blood of the animals receiving the diets with mealworm meal might further support this hypothesis. Eosinophilia can be indicative of an allergic reaction, but also of a variety of diseases, like parasitic infections, neoplasia, gastrointestinal disorders, and others [52]. However, as the number of eosinophilic granulocytes was within the reference range of healthy individuals, and as the dogs of the present study did not show any clinical signs of a food allergy, the pathophysiological relevance of the present findings seems to be questionable.

Likewise, the observed effects of the diets on the phagocytic activity of blood granulocytes as well as on the concentrations of IgA and IgE in the plasma of the dogs were only small. An interaction between the dietary protein source and protein level could be detected for these immune variables, which was, however, not further clarified by the consecutive group comparisons carried out. In general, phagocytizing granulocytes are a component of the innate immune system, while Igs are produced by B cells as an integral part of the adaptive immune response [37]. The different classes of Igs are linked with specific effector functions. For instance, IgA is involved in neutralization and opsonization of antigens as well as in complement activation, while IgE is especially relevant for mast cell sensitization [37]. Although the latter is an important underlying mechanism for type I hypersensitivity [53], it has been demonstrated that the detection of food-specific IgE in the plasma and saliva of dogs was not adequate for the diagnosis of adverse food reactions [54]. In the present study, only total plasma IgE concentrations were measured, while food-specific IgE was not assessed. Moreover, since the interaction effect between the dietary protein source and protein level could not be further elucidated, the findings on the plasma IgE concentrations appear to be of limited clinical relevance.

Nevertheless, our data demonstrate that mealworm meal in a diet differently modulated the immune response in healthy dogs than poultry meal, as a more conventional dietary protein source. At this point, it can only be speculated which component(s) of the mealworm meal might have mediated the immunological effects observed. On the one hand, it should be taken into consideration that the dogs of the present study did not receive a diet with mealworm meal before. Thus, mealworm antigens were unknown to the immune system of the animals, which could have induced a specific immune response. On the other hand, edible insects show a different nutrient profile than meat, for instance concerning their mineral, amino acid, and fatty acid concentrations [55]. The fatty acid patterns of the diets were not analyzed in the present study, but the amino acid profiles and mineral contents were. As different mineral supplements were used to balance the diets, the concentrations of macro and trace elements were comparable among all groups. However, the amino acid patterns differed as expected. The latter might be a potential explanation for the observed dietary effects on the immune system. Amino acids, such as arginine, glutamine, alanine, or proline, have been demonstrated to impact the immune function [56, 57], although specific studies in dogs are missing. Another dietary factor that could have modulated the immune response in the dogs of this study is the chitin content of the mealworm meal. Chitin is a polysaccharide present in the exoskeleton of insects [58]. The chitin content of insect meals varies [58] but has been demonstrated to range between 4.92 and 13.0% in *Tenebrio molitor* larvae [59]. Dogs only have a low intestinal chitinase activity [60], so the digestion of chitin is limited. Thus, undigested chitin could have potentially mediated specific immunological effects in the dogs of the present study. Chitin shows anti-inflammatory properties, as recently summarized by Wijesekara and Xu [61]. Those effects can be directly related to an interaction of chitin with the immune system [61]. In addition, an indirect impact by an interplay with the gut microbiome might also be possible, as already discussed for the chitin derivative chitosane [62]. Nevertheless, studies on the immunomodulatory properties of chitin are missing in dogs so far, and a potential link to the affected immune variables in the dogs of the present investigation fed the diets with mealworm meal remains speculative at this point.

With regard to the presence of indigestible carbohydrates in the experimental diets, it should also be critically discussed that the feed analyses that could be carried out in this study only allowed for a quantification of crude fiber. A more precise determination of different dietary carbohydrate fractions is the analysis of neutral detergent fiber, acid detergent fiber, total dietary fiber, or nonstarch polysaccharides [63]. Further laboratory feed analyses would have facilitated the discussion on a potential contribution of specific dietary fiber fractions to the observed immunological effects, and should therefore be considered in future investigations on this topic.

It should finally be noted that not only the dietary protein source, but also the protein level, partly influenced the immune system of the dogs in this study. In general, the importance of an adequate protein supply for the immune function of dogs is well known [25, 64]. However, investigations on the interaction between the overall protein supply and the canine immune system remain scarce [65, 66]. Current immunological studies are especially focused on different dietary protein sources [9, 15, 67–69] and hydrolyzed protein diets [70–73]. Variations in the dietary protein level have been investigated with regard to an impact on the fecal microbiota of dogs [74–83], while results on the immune function are widely missing. Thus, for future studies and to compare the data of the present work, it would be interesting to consider also graduated protein concentrations in the diet for immunological evaluations in dogs.

There are some limitations of this study, which should be considered for the data interpretation. The statistical analysis had to be carried out with an unequal group size, as 4 dogs refused the moderate-protein diet based on poultry by-product meal. The reason for the low acceptance of this diet remains unclear, especially as the high-protein diet with the same ingredients was well accepted by all animals. An appropriate statistical model was used to compensate for the missing dogs in the data set; however, a careful interpretation of the results is recommended.

Dogs of both sexes were included in our investigation. As only limited information is available if the sex or reproductive status impacts the immune system of canines [84–87], it would have been interesting to compare the dietary effects also between male and female dogs. However, due to the small sample size, a separate statistical evaluation was not possible. Nevertheless, as the dogs received each diet, they served as their own control, which also compensated for a potential influence of the sex of the animals on the present results.

Furthermore, it should be noted that non-allergic dogs were used in this study, as we aimed to evaluate the immunomodulatory properties of mealworm meal in a diet in healthy organisms. However, insect-based diets are also an interesting therapeutic option for animals suffering from a food allergy [8–10, 21]. The present results should not be transferred to allergic patients without hesitation. A food allergy is characterized by a compromised immunotolerance to dietary allergens [88]; thus, the immune function of healthy and allergic dogs differs. As it can be assumed that the effects of dietary interventions vary depending on the immune status of the animal, further studies are required to evaluate the immunological impact of diets with mealworm meal also in diseased dogs.

In this study, mealworm meal was chosen as an insect meal for the experimental diets. Since insect species differ in their nutritional composition [89], the immune response of the host to insect meals might also vary. Therefore, comprehensive evaluations of the immunological effects of different kinds of insect-based diets in dogs are recommended. In this context, the present investigation provides valuable insights into the immunomodulatory properties of mealworm meal, an insect meal that has not been extensively studied in dogs so far.

## Conclusions

The dietary inclusion of mealworm meal partly influenced the cellular and humoral immune response of healthy adult dogs. Besides, the dietary protein level also revealed an effect on selected immune variables of the animals. As all dogs remained healthy throughout the study, the clinical relevance of these findings, however, seems to be small. Research with further insect meals is required to conclude on their immunological effects in healthy and diseased dogs in general. Overall, considering the marked differences in the protein supply in practice, one should be aware of the immunomodulatory potential of such dietary variations.

## Data Availability

The datasets analyzed during the current study are available from the corresponding author on reasonable request.
